# Effects of Guizhi decoction for diabetic cardiac autonomic neuropathy: A protocol for a systematic review and meta-analysis

**DOI:** 10.1097/MD.0000000000022317

**Published:** 2020-09-25

**Authors:** Junmin Chen, Jiawei Cai, Mengya Wei, Xiaoran Zhang, Min Zhong, Min Liu, Yang Yu, Qiu Chen

**Affiliations:** a Hospital of Chengdu University of Traditional Chinese Medicine, Chengdu; b College of acupuncture and massage, Chengdu University of Traditional Chinese Medicine , No 37 Shi-er-qiao Road, Chengdu, Sichuan Province, PR China.

**Keywords:** diabetic cardiac autonomic neuropathy, Guizhi decoction, meta-analysis, protocol, systematic review

## Abstract

**Background:**

Diabetic cardiac autonomic neuropathy (DCAN) is one of the serious complications of diabetes. The pathogenesis of DCAN has not been fully elucidated. There is currently no effective treatment for such chronic disease. Traditional Chinese medicine has a long clinical history for the prevention and treatment of diabetes and chronic complications, and it also shows certain advantages in the treatment of DCAN. Many clinical studies have confirmed that Chinese medicine Guizhi decoction can reduce the clinical symptoms and improve neuronal function of patients with DCAN. So we intend to conduct a systematic review further clarified the effectiveness and safety of Guizhi decoction for DCAN.

**Methods:**

We will search each database from the built-in until July 2020. The English literature mainly searches Cochrane Library, PubMed, EMBASE, and Web of Science, while the Chinese literature comes from CNKI, CBM, VIP, and Wangfang database. Simultaneously we will retrieval clinical registration tests and grey literatures. In this study, only the clinical randomized controlled trials (RCTs) were selected to evaluate the efficacy and safety of Guizhi decoction in the treatment of DCAN. The 2 researchers independently conducted literature selection, data extraction, and quality assessment. Statistical heterogeneity among studies will be evaluated using the Cochran Q test (x^2^) and the *I*^2^ statistical value. We will utilize the Review Manage software V5.3.0 (The Nordic Cochrane Center, The Cochrane Collaboration, 2014, Copenhagen, Denmark) to statistically analyze all data.

**Ethics and dissemination:**

This study is a protocol for a systematic review of Guizhi decoction as a treatment of DCAN patients.

**Results:**

This study will provide high-quality synthesis of effectiveness and safety of Guizhi decoction for DCAN.

**Conclusion:**

This systematic review aims to provide new options for Guizhi decoction treatment of DCAN in terms of its efficacy and safety.

**Registration number:**

INPLASY202080018.

## 1 Introduction

Cardiac autonomic nerves (CANs) include the sympathetic nerve and the vagus nerve, which check and balance each other and play important roles in the regulation of heart rate, conduction, and cardiac contractility. Diabetic cardiac autonomic neuropathy (DCAN) is one of the severe complications of diabetes mellitus; damage of CANs in a high glucose environment causes sympathetic-vagal imbalance,^[1,2]^ which thus causes abnormal neurotransmitter signaling and increased asynchronization of cardiac electrophysiology.^[3,4]^ About 2.5% to 50.0% of diabetic patients have cardiac autonomic neuropathy.^[5]^ It is reported that diabetic cardiac autonomic neuropathy is easy to be ignored in many complications of diabetes. Because the balance of cardiac autonomic nerves is broken, the incidence of myocardial ischemia, painless myocardial infarction and sudden cardiac death is increased,^[6]^ and the mortality rate within 5 years is as high as 50%, of which sudden cardiac death accounts for 28%.^[2,7,8]^ Compared with the patients without DCAN, the mortality increased 3 to 4 times.^[9,10]^ The time variability of left ventricular repolarization in DCAN patients increased, and the proportion of malignant arrhythmia caused by it in the cause analysis of sudden death was 50% to75%.^[11]^ According to a research, strict blood sugar control could slow the progression of CAN diseases but could not reverse CAN pathological changes.^[12]^ Therefore, in addition to protecting the sympathetic and vagus nerves from further damage, regulation of the imbalance of these 2 nerves is even more important for the treatment of diabetic CAN diseases. Because the pathogenesis of the disease has not been fully elucidated, western medicine lacks the corresponding treatment plan, some drugs interfere with glycolipid metabolism, drug target is single, the safety, and effectiveness need to be further confirmed.^[13]^ Our previous studies have shown that based on the treatment methods of balancing yin and yang and harmonizing Ying and Wei in Chinese medicine theory, taking Xinhe granule and Tiaoxin decoction containing Guizhi decoction can improve the symptoms of autonomic neuropathy of coronary heart disease.^[14,15]^ Traditional Chinese medicine suggests that diabetic cardiac autonomic neuropathy belongs to the category of “chest obstruction” and “palpitation”. Guizhi decoction can effectively play the role of regulating Ying Wei, reverse the remodeling of myocardial collagen, inhibit inflammatory factors, inhibit the abnormal remodeling of sympathetic nerve, and maintain the balance of autonomic nerve.^[16]^ Studies also confirmed that Guizhi decoction decreased the accumulation of inflammatory factors including nuclear factor κ B (NF-κ B), interleukin-1 (IL-1), tumor necrosis factor-α (TNF-α), and endothelin (ET-1) in the heart of streptozocin (STZ) rats; reversed spontaneous myocardial collagen remodeling in diabetic rats; significantly reduced myocardial basement membrane thickness; prevented damage and thickening of the myocardial basement membrane; and improved oxygen diffusion barriers,^[17,18]^ thus to prevent and treat diabetic myocardial damage.

In recent years, the advantages of traditional Chinese medicine in the prevention and treatment of this kind of chronic diseases have been widely recognized around the world.^[19]^ The prescription of Guizhi decoction firstly recorded in the book of Shanghan Lun written by Zhang Zhongjing, is composed of Ramulus Cinnamomi (Guizhi), Paeonia lactiflora (Shaoyao), Rhizoma Zingiberis Recens (Shengjiang), Licorice (Gancao), and Jujube (Dazao). We intend to collect randomized controlled trials (RCTs) about Guizhi decoction for DCAN based on the basis of evidence-based medicine, and conduct a meta-analysis of its efficacy and safety to provide higher quality clinical evidence for Chinese medicine treatment of DCAN.

## 2 Methods

### 2.1 Protocol registration

The systematic review protocol has been registered on the LNPLASY website as INPLASY202080018. (https://inplasy.com/inplasy-2020-8-0018/). It is reported following the guidelines of Cochrane Handbook for Systematic Reviews of Interventions and the Preferred Reporting Items for Systematic Reviews and Meta-analysis Protocol (PRISM).^[20]^

### 2.2 Eligibility criteria

#### 2.2.1 Study design

This study only included RCTs of Guizhi decoction for DCAN. However, animal experiments, reviews, case reports, and non-randomized controlled trials are excluded.

#### 2.2.2 Participants

DCAN patients must meet the World Health Organization (WHO) “diabetes” diagnosis standard in 1999.^[21]^ The diagnosis standard of cardiac autonomic neuropathy shall refer to Toronto standard and European cardiovascular disease prevention guidelines,^[22,23]^ regardless of race, gender, and age. Special patients with severe diabetic complications, severe cardiac insufficiency, pregnancy, etc. are not included.

#### 2.2.3 Interventions

Both groups were cured with conventional diabetes treatments recommended by the American Diabetes Association (ADA) guidelines, including diet, exercise, and hypoglycemic, and lipid-lowering therapies.^[24]^ The experiment group used Guizhi decoction or modified Guizhi decoction, while the control group applied for placebo, nutritional neurological drugs, or no treatment. In addition, the 2 groups did not take any drugs that interfered with the outcome indicators. Trials will be included at least 4 weeks of treatment.

#### 2.2.4 Outcomes

The primary outcomes include patient before and after treatment: markedly effective: symptoms improved significantly >70%; effective: symptoms reduced by 30% to 70%; ineffective: symptom improvement is <30% or no improvement, or even worse. In addition to Heart rate variability (HRV) recognized as the most accurate and sensitive index to judge whether diabetic patients have autonomic nervous system damage, fasting blood glucose (FBG) and 2 hours Postprandial Blood Glucose (2 hours PBG) were also included. Secondary outcomes included glycosylated hemoglobin and adverse events.

### 2.3 Study search

We will search each database from the built-in until July 2020. The English literature mainly searches Cochrane Library, PubMed, EMBASE, and Web of Science, while the Chinese literature comes from CNKI, CBM, VIP, and Wangfang database. Simultaneously we will retrieval clinical registration tests and grey literatures. According to the PICOS principle, the keywords of our search terms were: (“Guizhi Tang” OR “Guizhi Decoction”) AND (“diabetic cardiac autonomic neuropathy” OR “DCAN”).

### 2.4 Study selection

**Figure 1:**
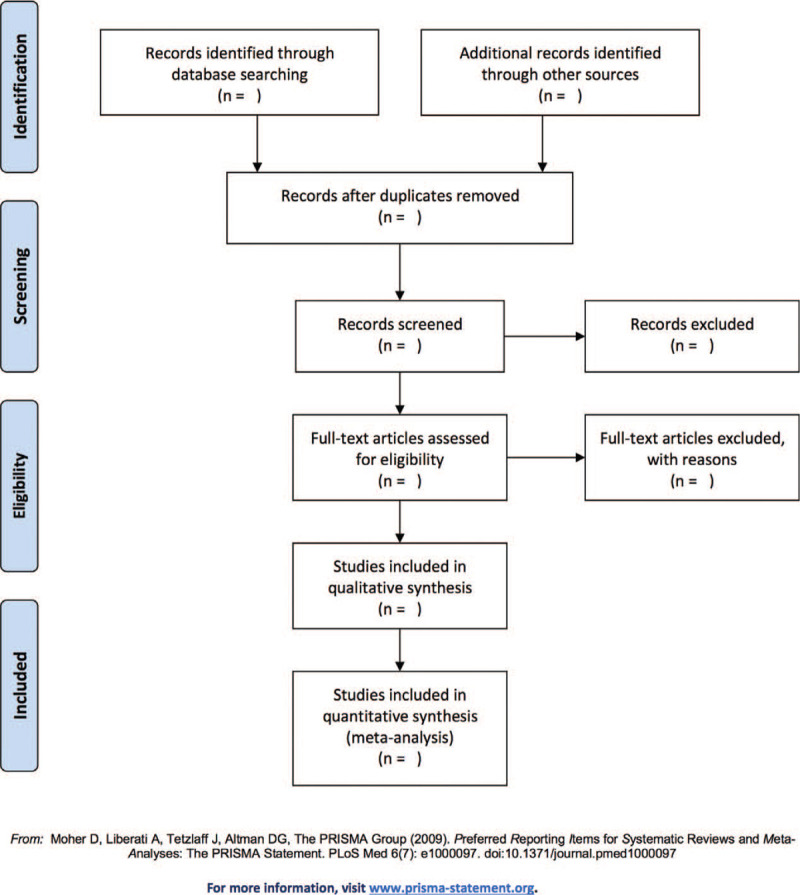
Flow diagram of study selection.

We will manage the electronic citations we downloaded from the above databases in Endnote X8 for Mac (Thomson Reuters, USA). First of all, 2 independent reviewers initially screened the literatures that did not meet the pre-established standards of the study by reading the title and abstract. Secondly, download the remaining literatures and read the full text carefully to further decide whether to include or not. Finally, the results were cross-checked repeatedly by reviewers. A final decision will be made through consensus when there were discrepancies. Details of the selection process were shown in the flow chart (Fig. 1).

### 2.5 Data extraction and management

According to the characteristics of the study, we prepared excel table for data acquisition before data extraction. The results of the qualified study were extracted independently by 2 reviewers and the data extraction form was filled in. Duplicate literature will be removed. If there is any dispute, the 2 reviewers can discuss or consult the third party to reach an agreement. The main data extracted are as follows: first author, year of publication, source of funds, intervention measures in experimental group, intervention measures in control group, treatment time, course of disease, number of patients in each group, age, gender, prognosis, and safety of patients. If you find something unclear in the study, you can contact the author of the communication directly for more detailed information. The above information was finally cross checked by 2 reviewers.

### 2.6 Risk of bias assessment

Two review authors will independently evaluate the design, execution, and reporting of the included RCTs based on the Cochrane risk of bias tool. The following 7 items affiliated with bias risk, including random sequence generation, allocation concealment, blinding of participants and personnel, blinding of outcome assessment, incomplete outcome data, selective outcome reporting, and other biases, will be evaluated by 2 reviewers. Each item is classified as “Low risk”, “High risk” or “Unclear risk”. The discrepancies will get a consistent conclusion by discussing between both reviewers or seeking the third-party consultation.

### 2.7 Statistical analysis

The risk ratio (RR) for dichotomous data will be calculated, respectively, along with 95% CI, the weighted mean difference (WMD). For continuous data, the mean difference (MD) or standardized mean difference (SMD) with 95% CI will be estimated. If we use the same scale to measure an outcome in different studies, we will use MD. Similarly, if we use different scales to measure the same outcome, we will use SMD. If an outcome measure contains less than 2 trials, we will summarize the results descriptively. Statistical heterogeneity among studies will be evaluated using the Cochran Q test (x^2^) and the *I*^2^ statistical value. We will categorize the heterogeneity using the following rules. *I*^2^ of 0% to 25% indicates low heterogeneity. *I*^2^ of 25% to 50% represents moderate heterogeneity. And *I*^2^ of 75% to 100% represents high heterogeneity. When the P value from a x^2^ test is more than .10 or *I*^2^ 50%, we will adopt the fixed-effects model. Otherwise, there will be perceptible differences between the studies. Subgroup analysis will be performed to identify possible explanations for statistical heterogeneity, taking into account pre-specified factors.

We will utilize the Review Manage software V5.3.0 (The Nordic Cochrane Center, The Cochrane Collaboration, 2014, Copenhagen, Denmark) to statistically analyze all data. The overall RR with its 95% CI for dichotomous data will be estimated. The MD or SMD with 95% CI will be calculated for continuous data in different situations. The fixed-effects model will be employed as appropriate for analysis. If the heterogeneity in the study is significant, subgroup analysis will be conducted to investigate possible sources of statistical heterogeneity. When a meta-analysis is not available, descriptive summaries of individual studies will be provided.

### 2.8 Additional analysis

#### 2.8.1 Subgroup analysis

If the results are heterogeneous, we will conduct subgroup analysis based on different reasons. Heterogeneity is manifested in race, age, gender, different intervention methods, drug dosage, course of treatment and so on.

#### 2.8.2 Sensitivity analysis

To identify the robustness of the meta-analysis results, we will conduct a sensitivity analysis by omitting each of the RCT, or excluding the RCTs with high risk of bias, or excluding the RCTs with missing data.

#### 2.8.3 Reporting bias

If there are more than 10 studies in meta-analysis, the symmetry of the funnel plot will be evaluated to check for publication bias and to interpret the results carefully, grading the quality of evidence. In this systematic review, the evidence quality of the whole study was assessed by the “recommended assessment, formulation and evaluation (grade)” standard developed by the World Health Organization and international organizations to achieve transparency and simplification. The quality of evidence was divided into 4 levels: high, medium, low, and very low. Slope profiler 3.2 will be used for analysis.

## 3 Discussion

DCAN is a common chronic complication of diabetes. At present, drug treatment is universally used in the treatment of this disease, and there are some shortcomings, such as little effect or large side effects. Therefore, both clinicians and patients hope to seek a new treatment to improve symptoms with low adverse reactions. Traditional Chinese medicine has been used to treat diabetes and diabetic complications in China for many years.^[25,26]^ It has not only outstanding efficacy, but also has few side effects and low economic costs. Clinical studies have shown that Guizhi Decoction can alleviate the symptoms of DCAN and improve the overall clinical efficacy.^[27–29]^ However, there is no evidence-based medicine to confirm the efficacy of Guizhi Decoction for DCAN. So we attempt to perform this meta-analysis to provide high-quality evidence for the clinical efficacy and safety of Guizhi Decoction. Finally, we will classify the existing evidence to provide a better guide for clinical use.

## Author contributions

**Conceptualization:** Junmin Chen, Jiawei Cai, Qiu Chen, Yang Yu.

**Data curation:** Mengya Wei, Xiaoran Zhang.

**Funding acquisition:** Qiu Chen, Yang Yu.

**Investigation:** Min Zhong, Min Liu.

**Methodology:** Junmin Chen, Jiawei Cai, Qiu Chen.

**Project administration:** Qiu Chen, Yang Yu.

**Software:** Junmin Chen, Jiawei Cai.

**Supervision:** Xiaoran Zhang.

**Validation:** Junmin Chen.

**Writing – original draft:** Junmin Chen, Jiawei Cai.

**Writing – review & editing:** Qiu Chen, Yang Yu.
